# Nucleus accumbens response to gains in reputation for the self relative to gains for others predicts social media use

**DOI:** 10.3389/fnhum.2013.00439

**Published:** 2013-08-29

**Authors:** Dar Meshi, Carmen Morawetz, Hauke R. Heekeren

**Affiliations:** ^1^Cluster of Excellence “Languages of Emotion,” Freie Universität BerlinBerlin, Germany; ^2^Department of Education and Psychology, Freie Universität BerlinBerlin, Germany; ^3^Dahlem Institute for the Neuroimaging of Emotion, Freie Universität BerlinBerlin, Germany; ^4^Berlin School of Mind and Brain, Humboldt Universität zu BerlinBerlin, Germany

**Keywords:** reputation, impression management, social reward, social media, Facebook, nucleus accumbens, individual differences, fMRI

## Abstract

Our reputation is important to us; we've experienced natural selection to care about our reputation. Recently, the neural processing of gains in reputation (positive social feedback concerning one's character) has been shown to occur in the human ventral striatum. It is still unclear, however, how individual differences in the processing of gains in reputation may lead to individual differences in real-world behavior. For example, in the real-world, one way that people currently maintain their reputation is by using social media websites, like Facebook. Furthermore, Facebook use consists of a social comparison component, where users observe others' behavior and can compare it to their own. Therefore, we hypothesized a relationship between the way the brain processes specifically self-relevant gains in reputation and one's degree of Facebook use. We recorded functional neuroimaging data while participants received gains in reputation, observed the gains in reputation of another person, or received monetary reward. We demonstrate that across participants, when responding to gains in reputation for the self, relative to observing gains for others, reward-related activity in the left nucleus accumbens predicts Facebook use. However, nucleus accumbens activity in response to monetary reward did not predict Facebook use. Finally, a control step-wise regression analysis showed that Facebook use primarily explains our results in the nucleus accumbens. Overall, our results demonstrate how individual sensitivity of the nucleus accumbens to the receipt of self-relevant social information leads to differences in real-world behavior.

## Introduction

Reputation can be defined as a person's overall quality of character as judged by others (Merriam-Webster et al., 2010). From an evolutionary perspective, we care about our reputation because it indicates that other community members can cooperate with us, and this cooperation provides us with greater access to resources, which in turn enhances survival rates (Alexander, 1987; Nowak and Sigmund, 1998; Wedekind and Milinski, 2000; Milinski et al., 2002). In other words, there has been natural selection driving us to form a good reputation in the eyes of others.

How we process information related to our reputation has recently been the topic of neuroscientific research (for review see Izuma, 2012). This research has demonstrated that the processing of gains in reputation (positive social feedback related to one's character) occurs in the ventral striatum (Izuma et al., 2008; Korn et al., 2012). Furthermore, discovering that others like us, or have provided general positive feedback about us, is also processed in the ventral striatum (Davey et al., 2010; Jones et al., 2011). The ventral striatum, which includes the nucleus accumbens, has been well-established in the processing of other rewards which motivate human behavior, such as money or food (for review see Haber and Knutson, 2010). Simply observing pictures of rewards can activate the nucleus accumbens, and recent studies have demonstrated that individual differences in the nucleus accumbens response to pictures of food or sex predicts either subsequent food consumption or sexual desire, respectively (Demos et al., 2012; Lawrence et al., 2012). Importantly however, no study has yet investigated how individual differences in the neural processing of self-relevant, social information may be related to differences in real-world social behavior, including more specifically, behavior aimed at obtaining a good reputation.

In today's world, we can obtain a good reputation in a variety of ways; for example, we can be polite to people or behave in a moral manner. We can also manage our reputation online via social media websites (Tennie et al., 2010). A majority of the interaction on social media websites is in public, visible to the user's group of friends. Hence, social media use inherently impacts one's reputation. In support of this, it has recently been demonstrated that people use social media websites for impression management (Krämer and Winter, 2008; McAndrew and Jeong, 2012), and to maintain and increase their social capital (Ellison et al., 2007). Moreover, social media websites such as Facebook, appear to encourage users to engage in self-promoting behavior (Buffardi and Campbell, 2008; Mehdizadeh, 2010; Ryan and Xenos, 2011) which has been linked to attempts at acquiring a good reputation (Baumeister, 1982; Bromley, 1993). Furthermore, another aspect of social media use is social comparison (McAndrew and Jeong, 2012). Inherent in using social media websites like Facebook is the observation of others' behavior, and importantly, the observation of the positive feedback they receive for their posts (e.g., “likes”). Thus, users are able to compare others' behaviors and feedback, to their own.

**Figure 1:**
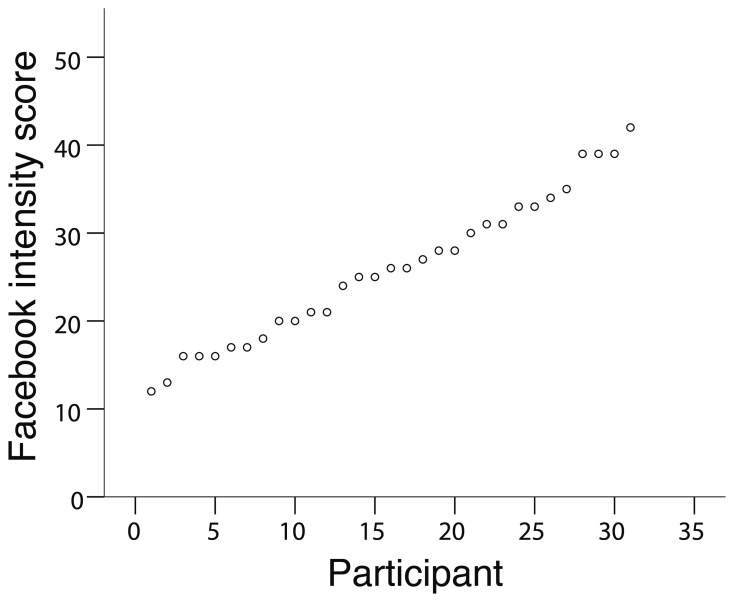
**Distribution of Facebook intensity scores across all participants**.

With social media's relation to reputation management in mind, we employed Facebook use as a proxy for a real-world behavior aimed at obtaining a good reputation. We selected participants for their Facebook use (Figure 1) and then, in the scanner, participants received gains in reputation, observed the gains in reputation of another person, or received monetary reward. We hypothesized that individual differences in the nucleus accumbens response to gains in reputation for the self, relative to observing gains for others (what we term “self-relevant”), will predict Facebook use (see fMRI data analysis in Materials and Methods). Conversely, nucleus accumbens sensitivity to monetary reward should not predict Facebook use because personal use of the Facebook website is not motivated by obtaining monetary reward.

## Materials and methods

### Participants

We recruited 31 healthy, right-handed participants (14 males) between 19 and 31 years of age (mean = 23.1, *SD* = 3.2). All participants had no history of psychiatric or neurological disorder and gave written informed consent in accord with local ethics. German was the native language of all participants (the experiment was conducted in German, although depicted in English).

### Experimental cover story

While being recruited, participants were told that the experiment would take place over two different days. Upon arriving for Day 1, participants were told that they would be given an on-camera interview and then asked to fill out a packet of questionnaires. Importantly, participants were told that between Day 1 and Day 2, the recorded video interview would be individually observed by 10 anonymous reviewers (5 males and 5 females). These reviewers, after seeing the video, would select 10–15 words from a list of 200 adjectives that they thought accurately described the participant. Of note, participants were told that the anonymous reviewers would not watch the video if they already knew the participant. The participants were then told that when they returned for Day 2 of the experiment they would learn what the reviewers thought of them, as well as perform a card choice task where they could earn money. Participants were told they would be paid 10 Euro for Day 1, and could win from 10 to 22 Euro by playing the card game on Day 2. This experimental cover story and paradigm is similar to a previously published experiment used to investigate the neural substrates involved in processing social reward (Izuma et al., 2008).

### Procedure

**Figure 2:**
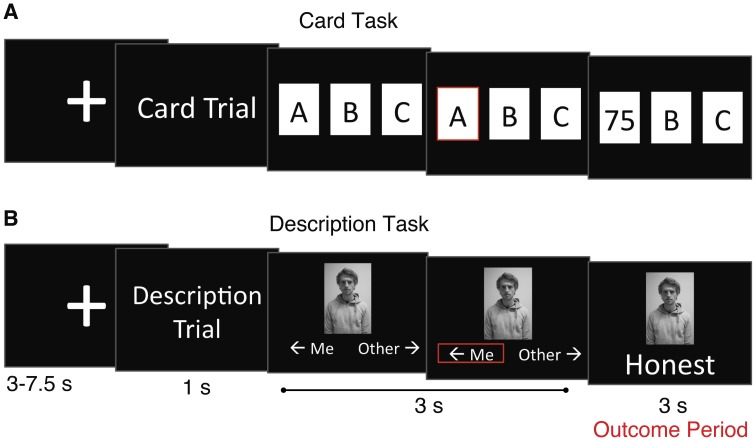
**The card and description tasks.** At the beginning of each trial, participants were shown a message indicating which type of trial they were about to perform (1 s). **(A)** In the card task, participants were presented with three cards and required to choose one (3 s; answers were confirmed by a red outline around the card). The monetary outcome of their choice was then revealed (3 s). They received either a High-win (75, 80, or 85 cents), a Low-win (25, 30, or 35 cents), or a No-win (“XX”) outcome. **(B)** In the description task, participants saw a picture of either themselves or of an “other” person who took part in the experiment (participants were told that this other participant had also completed the on-camera interview and then received feedback). Participants were required to indicate the identity of the person in the picture (3 s; answers were confirmed by a red outline). A word was then displayed below the picture (3 s). Participants believed that this word was selected by the anonymous reviewers to describe the person in the picture. Importantly, the anonymous reviewers did not exist; they were only part of the experimental cover story. In reality, the participants received either a pre-determined range of positive feedback concerning their reputation, a pre-determined range of positive feedback concerning the “other” participant's reputation, or participants saw “xxxxxxxx,” which was used as a No-feedback control for both the self and other conditions (see Materials and Methods).

During recruitment, participants completed the Facebook Intensity Scale in an online format (for actual scale, see below) (Ellison et al., 2007). Upon arriving on Day 1, participants were briefed about the experimental procedure and then interviewed. The on-camera interview was recorded by a digital camera (FujiFilm, Japan) which was set up on a tripod. Participants were asked to briefly introduce themselves and then answered a series of questions (see below). Interviews lasted around 10–15 min. After the interview, a digital picture of the participant was taken, to be used in the experimental display during Day 2 (see Figure 2). Participants then filled out several questionnaires (see below). In addition to these surveys, participants were also asked to sign a release waiver for the video. This release waiver appeared to grant legal access to the 10 anonymous reviewers so they could watch the participant's video.

Upon arriving for Day 2, participants were given instruction and training on the experimental tasks they would perform. To note, participants returned after an average of 20.4 days (*SD* = 7.0; range = 10–32 days). The training consisted of one example trial of each task condition described below (card task, description task self, description task other; all training outcomes were No-win/No-feedback; see below).

In the scanner participants performed two different tasks, a card task (for monetary reward) and a description task (for positive social feedback), in an event-related manner (Figure 2). At the start of each trial, participants saw a fixation cross for a variable amount of time per trial (3–7.5 s with display times following an exponential distribution with most times at the lower end of the range, mean display time of 4 s). They then discovered which task they would be performing in that trial by a 1 s display (either “Card Trial” or “Description Trial”). In a card task trial, participants were presented with three cards (A, B, or C) and they had 3 s to choose one. Participants responded using a three-button panel with the buttons horizontally aligned to match the three options. After choosing a card, a red outline appeared around the card to visually confirm their choice. The next display informed them of the monetary outcome of their choice for 3 s. On each card trial they experienced 1-of-3 different monetary outcomes: (1). High-win (75, 80, or 85 cents), (2). Low-win (25, 30, or 35 cents), (3). No-win (“XX”). In addition, they were not told before scanning that there would be different categories of monetary rewards.

In a description trial, participants were presented with a black-and-white picture of either themselves or an “other” participant. Participants were told that this other participant was someone who had also completed the on-camera interview and then received feedback while in the scanner. To note, participants had never seen the other participant before, and the other participant was gender matched to the current participant (male with male, female with female; only one male, and one female picture were used as the other participant for the entire experiment). Participants were required to identify the person in the picture by pressing either left or right on the three-button panel as indicated on the display (see Figure 2). The left and right options were held constant for each participant over the course of the experiment, and direction was counterbalanced across participants. After identifying the person in the picture, a red outline appeared around the word to visually confirm their choice. The next display revealed a word below the picture that participants believed was chosen by one of the ten anonymous reviewers to describe the person in the picture. Importantly, the anonymous reviewers did not exist; they were only part of the experimental cover story. In reality, the words seen by the participants were 1-of-3 different types: (1). High-positive feedback, (2). Low-positive feedback, (3). No-feedback (“xxxxxxxx”; the mean number of letters in the entire set of word stimuli was 8.2, therefore 8 x's were used for the No-feedback condition). Therefore, because reputation is defined as a person's overall quality of character as judged by others, the participants received varying levels of positive social feedback regarding their reputation. In order to display positive feedback stimuli with the high and low split, words were taken from a previously established German word set in which native speakers rated word desirability on a 1–7 Likert scale, with the higher number being more desirable (Grühn and Smith, 2008). Example words translated from German are Cheerful, Intelligent, Relaxed, Honest, Moral, Direct, Proud, and Sentimental. Before scanning, words were divided into two levels (High-positive, Low-positive), and two sets of words were assembled at each desirability level, one for self-feedback and one for other-feedback. Therefore, there was no overlap of feedback words between the self and other conditions. To note, participants were not told before scanning that they would see words with different desirability ratings; they only experienced them as real feedback from the 10 reviewers.

Importantly, the above-mentioned desirability ratings and the split between high- and low-positive words accomplished before the experiment were not used for analysis. After the scanning session participants rated the desirability of all words (both the feedback they received for themselves and the words used to describe the other participant) by answering the question, “How good or bad is it for me, when I am …?” on a 1–7 Likert scale, with the higher number being more desirable. These individualized word desirability ratings were used in the analysis (see Trial Analysis below for more detail). Participants were then debriefed and paid 32 Euro.

### Trial analysis

Participants experienced 20 trials of each monetary outcome and social feedback condition resulting in 180 total trials (20 × 3 monetary outcomes, 20 × 3 self-feedback, 20 × 3 other-feedback). All conditions (monetary outcomes and social feedback source and levels) were proportionately distributed between the four different scanning runs, and randomized within each run. In each of these trials participants were required to respond, in the card task by choosing a card and in the description task by identifying the person in the picture. One hundred percent of trials were answered, and thus all 180 trials for each participant were used in analysis, except for one participant where technical difficulties during scanning allowed for only 135 trials to be analyzed.

As mentioned above, after scanning, participants rated the desirability of all descriptive words seen during the experiment (both the feedback they received for themselves and for the other participant). For analysis, description trials for each participant were classified according to their individual, post-experiment word desirability ratings. Specifically, in each condition (self-feedback and other-feedback), trials were rank ordered by the word's desirability rating and a median split was performed, which re-organized the trials into two groups, High-positive feedback, and Low-positive feedback. This median split was conducted to make the social reward conditions in the description task (High- and Low-positive feedback) analogous to the monetary reward conditions in the card task (High- and Low-win).

### Facebook intensity scale

During recruitment, before Day 1, participants completed the Facebook Intensity Scale in an online format (Ellison et al., 2007). This questionnaire assesses the degree to which people use and rely upon the online social networking platform, Facebook. The eight individual items on the Facebook intensity questionnaire, including the scoring scale, were as follows:

1. How many total Facebook friends do you have?
  – 10 or less friends = 0
  – 11–50 friends = 1
  – 51–100 friends = 2
  – 101–150 friends = 3
  – 151–200 friends = 4
  – 201–300 friends = 5
  – 301–400 friends = 6
  – 401–600 friends = 7
  – 601–800 friends = 8
  – 801–1000 friends = 9
  – 1001 or more friends = 10
2. In the past week, on average, approximately how many minutes per day have you spent on Facebook?
  – less than10 min = 1
  – 10–30 min = 2
  – 31–60 min = 3
  – 1–2 h = 4
  – 2–3 h = 5
  – more than 3 h = 6
  Participants then indicated how much they agreed with the following statements on a Likert scale (1 = Strongly disagree to 5 = Strongly agree). Scale rating was used in scoring:
3. Facebook is part of my everyday activity
4. I am proud to tell people I am on Facebook
5. Facebook has become part of my daily routine
6. I feel out of touch if I haven't logged onto Facebook for a while
7. I feel I am part of the Facebook community
8. I would be sorry if Facebook shut down

Facebook intensity scores were computed by adding responses for all eight items. A total of 84 potential participants filled out the Facebook Intensity Scale during recruitment. We selected the 31 participants in our study to have a broad range of Facebook intensity scores (min = 12, max = 42, mean = 25.9, *SD* = 8.4; see Figure 1). We also selected participants to balance gender across Facebook intensity scores (males: mean = 26.8, *SD* = 8.0; females: mean = 25.1, *SD* = 8.8). Analysis with a *t*-test revealed that there was no difference in Facebook intensity score with respect to gender [*t*_(29)_ = 0.546, *p* = 0.589]. No other criteria besides Facebook intensity score, gender and the previously noted aspects of the study mentioned above (see Participants section) were used to select participants. To note, the Facebook Intensity Scale has high reliability; analysis of our 31 participants' responses revealed a Cronbach's α of 0.905. In addition, participants had been using Facebook for an average of 35.1 months (*SD* = 14.6; range = 9–61 months).

### On-camera interview

For the on-camera interview which took place on Day 1 of the experiment, participants were asked to briefly introduce themselves and then answered the below questions:

1. Do you like living in Berlin?
2. Why did you choose your field of study/profession?
3. Please pick one problem facing modern German society and briefly state your opinion on the matter.
4. What is one of your proudest achievements?
5. What do you like to do in your free time? What are your hobbies?
6. Please think of a favorite creative work, such as a film, book, song, or artwork. What is it and why do you like it?
7. What would you do if you won 1 million euro in the lottery?
8. Where do you see yourself in 10 years?

### Personality surveys

After the interview on Day 1, participants filled out the following questionnaires:

1. Rosenberg Self Esteem Scale (Rosenberg, 1965)
2. Reynolds Social Desirability Scale (form C) (Reynolds, 1982)
3. Narcissistic Personality Inventory (16 question) (Ames et al., 2006)
4. Mehrabian Conformity Scale (Mehrabian and Stefl, 1995)
5. Beck Depression Inventory-II (BDI-II) (Beck et al., 1996a,b). Of note, no participant had a BDI-II score above 13.

We examined whether Facebook use correlated with any of the assayed personality measures. Facebook intensity positively correlated with self-esteem (Pearson's *r* = 0.364, *p* = 0.044) and narcissism (Pearson's *r* = 0.381, *p* = 0.035). No correlation was revealed between Facebook intensity and conformity (Pearson's *r* = 0.232, *p* = 0.208), depression-related affect (Pearson's *r* = 0.108, *p* = 0.563), or social desirability (Pearson's *r* = −0.303, *p* = 0.098). Of note, our narcissism result replicates previous behavioral research (Buffardi and Campbell, 2008; Mehdizadeh, 2010).

### fMRI acquisition

Scanning was performed at the Dahlem Institute for Neuroimaging of Emotion at the Freie Universität Berlin, Germany using a 3T Siemens Trio scanner (Siemens Healthcare Diagnostics GmbH, Erlangen, Germany). Stimuli were presented using the Cogent 2000 toolbox (http://www.vislab.ucl.ac.uk/cogent.php) for MATLAB (The Mathworks Inc.) on LCD-goggles (Resonance Technology Inc., Northridge, California). Anatomical images were acquired using a T1-weighted MPRage protocol (256 × 256 matrix, 176 sagittal slices of 1 mm thickness). Fieldmaps were acquired using a dual echo 2D gradient echo sequence with echos at 4.92 and 7.38 ms, and a repetition time of 488 ms. Functional images were acquired as echo-planar T2^*^-weighted images (repetition time = 2.0 s, echo time = 30 ms, matrix = 64 × 64, flip angle = 70°, field of view = 192 mm, interslice gap = 0.3 mm). A total of 37 oblique-axial slices (3 × 3 × 3 mm^3^ voxels) parallel to the anterior commissure-posterior commissure line were collected per volume. A total of 252 volumes were collected per experimental run, with 4 runs per participant.

### fMRI data analysis

FMRIB Software Library (FSL, version 4.1.9) (Smith et al., 2004) was used for fMRI data analysis on the High-Performance Computing system at Freie Universitaet Berlin (http://www.zedat.fu-berlin.de/Compute). Brain matter in the T1-weighted anatomical image was segmented from non-brain using a mesh deformation approach (Smith, 2002). Functional data were preprocessed using FSL default options: motion correction was applied using rigid body registration to the central volume (Jenkinson et al., 2002); Gaussian spatial smoothing was applied with a full-width half-maximum of 6 mm; high-pass temporal filtering was applied using a Gaussian-weighted running lines filter, with a cut-off of 100 s. Susceptibility-related distortions were corrected as far as possible using FSL fieldmap correction routines (Jenkinson, 2003). To address our hypothesis concerning the neural processing of monetary reward and positive social feedback, and their relation to Facebook use, a general linear model was fit to the data with the following 14 regressors (GLM 1):

– R1. When participants discovered the trial type (card or description task)

For the card task:

– R2. When participants were presented with the cards and made a choice
– R3. Outcome was High-win
– R4. Outcome was Low-win
– R5. Outcome was No-win

For the description task:

– R6. When participants were presented with their picture and answered accordingly
– R7. When participants were presented with a picture of the “other” participant and answered accordingly
– R8. Outcome for self condition was High-positive feedback
– R9. Outcome for self condition was Low-positive feedback
– R10. Outcome for self condition was No-feedback
– R11. Outcome for other condition was High-positive feedback
– R12. Outcome for other condition was Low-positive feedback
– R13. Outcome for other condition was No-feedback
– R14. Error trials (see Trial Analysis).

**Figure 3:**
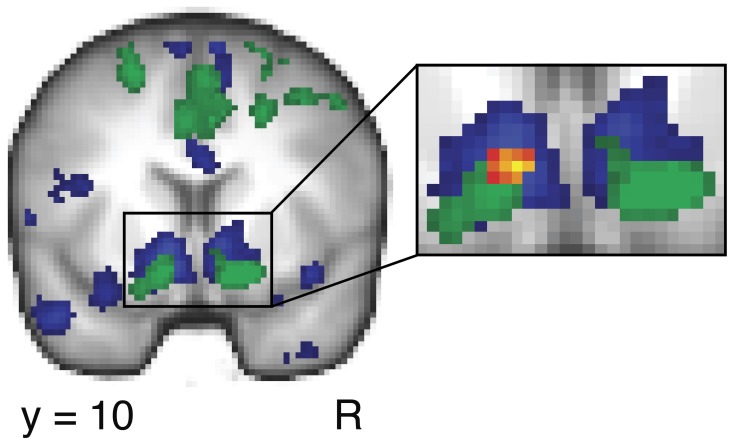
**Neuroimaging results demonstrating that Facebook use is predicted by the nucleus accumbens response to self-relevant gains in reputation across participants.** The monetary reward contrast (High-win > Low-win in card task) is depicted in blue, and the positive social feedback interaction contrast (Self High-positive feedback > Other High-positive feedback) > (Self Low-positive feedback > Other Low-positive feedback) is depicted in green. The results demonstrate an overlap between monetary and social reward conditions within the ventral striatum. BOLD activation maps thresholded at *Z* > 2.3, *p* < 0.05, cluster corrected. *Box*, Closeup of ventral striatum activity. Analysis at the group level using individual Facebook intensity scores and the positive social feedback interaction contrast was performed within a mask of the bilateral nucleus accumbens. The results demonstrate significant activity within the left nucleus accumbens, shown in red-yellow. BOLD activation thresholded at *Z* > 2.3, *p* < 0.05, cluster corrected for bilateral nucleus accumbens. R, right hemisphere.

All regressors were constructed as boxcar functions spanning the duration of the stimulus (R1 = 1 s; R2–14 = 3 s), and convolved using a double-gamma hemodynamic response function. Individual contrast images were computed and taken to a group-level mixed-effect analysis using voxel-wise one-sample *t*-tests. To determine the neural substrates involved in processing monetary reward, we performed a whole-brain, High-win > Low-win (R3 > R4) contrast for trials in the card task (Figure 3). To determine the neural substrates specifically involved in processing self-relevant, positive social feedback, we performed a whole-brain interaction contrast, (Self High-positive > Other High-positive) > (Self Low-positive > Other Low positive) [(R8 > R11) > (R9 > R12)] (Figure 3). This revealed changes in blood oxygen level-dependent (BOLD) signal due to differences between high and low positive feedback, specific to the self (see Contrasts to reveal gains in reputation, below). For both the monetary reward contrast and the social feedback interaction contrast, *Z*-statistic images were thresholded with default FSL cluster correction for multiple comparisons with a minimum *Z*-score set at 2.3 and a significance level set at *p* < 0.05.

To address our research question concerning the relation between the neural processing of positive social feedback and the degree of Facebook use, we looked for BOLD signal change specifically within the bilateral nucleus accumbens. To explain, the nucleus accumbens has been well established, in both animal and human studies, to be involved in processing different types of reward (for review see Haber and Knutson, 2010). In addition, a recent meta-analysis of 1351 different publications used Bayesian statistics to demonstrate that if the nucleus accumbens is active during a task, there is a 90% chance that the task is a reward task (Ariely and Berns, 2010). Importantly, recent research has demonstrated that the ventral striatum, which contains the nucleus accumbens, is active when a person receives positive social feedback concerning their reputation (Izuma et al., 2008). Finally, recent studies have demonstrated that individual differences in the nucleus accumbens response to pictures of food or sex predicts either subsequent food consumption or sexual desire, respectively (Demos et al., 2012; Lawrence et al., 2012). Therefore, due to the above evidence, we had the *a priori* hypothesis that there would be a relation, specifically in the nucleus accumbens, between an individual's degree of Facebook use and the neural processing of positive social feedback concerning one's reputation. Thus, for this analysis of our neuroimaging data, we entered each participant's Facebook intensity score as a covariate in the group level fMRI analysis. This covariate was orthogonalized with respect to the main effect group regressor in order to capture only linear parametric variance in the imaging data specifically due to Facebook use. To perform this covariate analysis, we set up a separate general linear model (GLM 2; identical to the last, except with this covariate at the group level). We created an anatomical region of interest mask of the bilateral nucleus accumbens from the Harvard-Oxford anatomical atlas (no minimum probability threshold, 2 mm resolution, 197 voxels). We then performed the monetary reward contrast (High-win > Low-win) and the social feedback interaction contrast (Self High-positive > Other High-positive) > (Self Low-positive > Other Low positive) within this mask with the covariate at the group level. *Z*-statistic images were thresholded using small volume FSL default cluster correction with a minimum *Z*-score set at 2.3 and a significance level set at *p* < 0.05.

To independently confirm and visualize our results from the above covariate analysis, we performed correlation analyses across participants between BOLD signal change and Facebook intensity scores. To avoid a potential non-independence error, we conducted a conjunction analysis independent of Facebook intensity score. The conjunction was performed between the social feedback interaction contrast (Self High-positive > Other High-positive) > (Self Low-positive > Other Low positive) and the monetary reward contrast (High-win > Low-win) using GLM 1 and restricted to the left and right sides of the nucleus accumbens (Harvard-Oxford anatomical atlas, no minimum probability threshold, 2 mm resolution, Left side = 111 voxels; Right side = 86 voxels). This conjunction analysis revealed a cluster of 69 voxels in the left nucleus accumbens (peak MNI coordinates −12, 12, −12; max *Z* = 3.32; *p* < 0.05, cluster corrected for left nucleus accumbens) and a cluster of 56 voxels in the right nucleus accumbens (12, 8, −12; max *Z* = 3.31; *p* < 0.05, cluster corrected for right nucleus accumbens). We then used these two clusters to create functional region of interest masks. From within these two masks, we extracted the parameter estimates from both the social feedback interaction contrast (GLM 1; Self High-positive > Other High-positive) > (Self Low-positive > Other Low positive) and the monetary reward contrast (GLM 1; High-win > Low-win). We then performed correlation analyses with these parameter estimates and the participants' individual Facebook intensity scores by calculating Pearson's correlation coefficient.

Furthermore, to confirm that there was no bias in this analysis due to more activation in either the social feedback interaction contrast or the monetary reward contrast, we compared the parameter estimates from each of these contrasts within the functional region of interest masks created by the conjunction analysis. In the left nucleus accumbens, the mean parameter estimate from the monetary reward contrast was 17.81 (*SD* = 20.60), and the mean for the social feedback interaction contrast was 18.51 (*SD* = 29.94). A paired *t*-test revealed no difference between the two groups of parameter estimates [*t*_(30)_ = −0.101, *p* = 0.920]. In the right nucleus accumbens, the mean parameter estimate from the monetary reward contrast was 17.94 (*SD* = 25.38), and the mean for the social feedback interaction contrast was 16.42 (*SD* = 26.20). A paired *t*-test revealed no difference between the two groups of parameter estimates [*t*_(30)_ = 0.212, *p* = 0.833].

### Contrasts to reveal gains in reputation

As we described above, we performed a social feedback interaction contrast (Self High-positive > Other High-positive) > (Self Low-positive > Other Low-positive) to isolate gains in reputation specific to self-relevant social reward. This contrast can also be written as (Self High-positive > Self Low-positive) > (Other High-positive > Other Low-positive), which illustrates that we isolated the difference between high-positive and low-positive gains in reputation for the self and subtracted the difference between observing high-positive and low-positive gains in reputation for another person. Another way to attempt to observe self-relevant social reward would be to perform the simple main effect contrast, Self High-positive > Self Low-positive, however, this would introduce a potential confound; the observed BOLD signal change could be explained by both differences in gains in reputation, and by simply observing variability in positive words. That is, seeing more positive words compared to less positive words may activate brain regions, irrespective of the relevance of these words to the participant, and in particular, her reputation. Therefore, to be sure that BOLD signal difference was a result of the association of positive social feedback to the participant, we made the *a priori* decision to include the “other” condition in our experiment and perform the social feedback interaction contrast. Notably, this is also the approach used by Izuma et al. (2008), where the authors also performed a social feedback interaction contrast using self and other feedback conditions to observe BOLD signal specific to receiving self-relevant positive social feedback.

We did explore the simple main effect contrast, Self High-positive > Self Low-positive, however. In the whole-brain, GLM 1 analysis, with a z-statistic threshold set at 2.7, this contrast revealed a significant cluster of 329 voxels in the striatum (16, 22, 0; max *Z* = 3.50; *p* < 0.05, cluster corrected). When we used each participant's Facebook intensity score as a covariate in the group level fMRI analysis and performed this contrast within a mask of the bilateral nucleus accumbens (GLM 2), no significant clusters were revealed.

### Regression analysis

**Figure 4:**
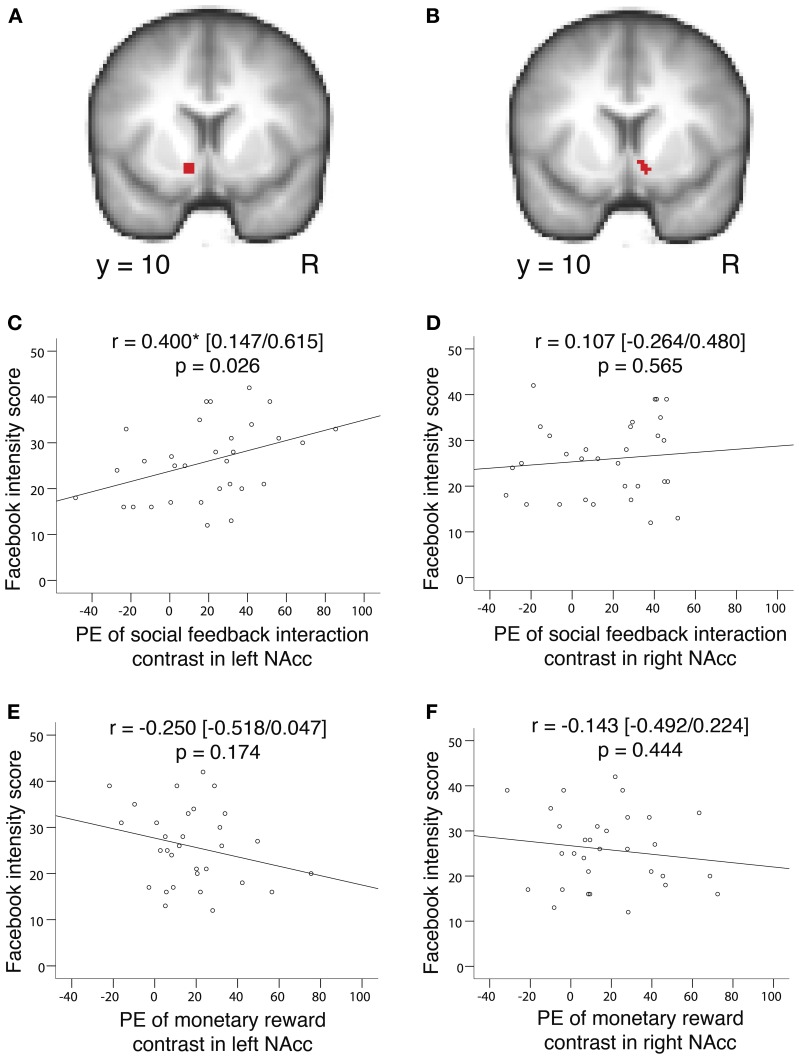
**Correlation analyses confirm that Facebook use is predicted by the left nucleus accumbens response to self-relevant gains in reputation across participants. (A,B)** Functional region of interest masks created by a conjunction analysis within the nucleus accumbens between the social feedback interaction contrast and the monetary reward contrast. To note, the conjunction analysis was performed independent of Facebook intensity scores. Correlation analyses between parameter estimates extracted from within these functional region of interest masks and Facebook intensity scores confirmed the result from our covariate analysis. **(C)** Activity in the left nucleus accumbens in response to positive social feedback regarding one's reputation, relative to observing positive social feedback for others, positively correlated with Facebook intensity scores (Pearson's *r* = 0.400, 95% confidence intervals = 0.147/0.615, *p* = 0.026). **(D)** Activity in the right nucleus accumbens in response to positive social feedback, relative to observing positive social feedback for others, did not correlate with Facebook intensity scores (Pearson's *r* = 0.107, 95% confidence intervals = −0.264/0.480, *p* = 0.565). **(E)** Activity in the left nucleus accumbens in response to monetary reward did not correlate with Facebook intensity scores (Pearson's *r* = −0.250, 95% confidence intervals = −0.518/0.047, *p* = 0.174). **(F)** Activity in the right nucleus accumbens in response to monetary reward did not correlate with Facebook intensity scores (Pearson's *r* = −0.143, 95% confidence intervals = −0.492/0.224, *p* = 0.444). PE, parameter estimate; NAcc, nucleus accumbens.

To confirm that individual differences in the left nucleus accumbens response are best explained by the degree of Facebook use rather than other personality traits, we performed a control step-wise regression analysis with the self-reported personality measures to predict the parameter estimates from the social feedback interaction contrast within the left nucleus accumbens (these parameter estimates were plotted in Figure 4C). The personality measures included in the analysis were the Facebook Intensity Scale, the Rosenberg Self Esteem Scale, the Reynolds Social Desirability Scale, the Narcissistic Personality Inventory, the Mehrabian Conformity Scale, and the BDI-II.

Two models were significant. The first demonstrated that Facebook use solely explains our results in the left nucleus accumbens [Adjusted *R*^2^ = 0.131, *F*_(2, 28)_ = 5.524, *p* = 0.026; Facebook intensity beta = 0.400]. The second demonstrated that Facebook use primarily, and conformity secondarily, explain our results in the left nucleus accumbens [Adjusted *R*^2^ = 0.325, *F*_(2, 28)_ = 8.227, *p* = 0.002; Facebook intensity beta = 0.510; conformity beta = −0.471].

## Results

### Whole-brain fMRI: monetary reward and gains in reputation

We examined changes in fMRI signal in the brain in response to monetary reward by examining the outcome period in the card task (see Figure 2). We performed a High-win > Low-win contrast and found significant changes in BOLD signal in the ventral striatum (peak MNI coordinates −10, 12, −6; max *Z* = 4.62; *p* < 0.05, cluster corrected; Figure 3 and Table 1).

**Table 1:** **Significant activation clusters for monetary reward**.

**Region**	**MNI coordinates**			**Cluster size**	**Peak *z***
	***x***	***y***	***z***		
**HIGH-WIN > LOW-WIN**					
L/R Ventral striatum	−10	12	−6	4821	4.62
L/R Anterior cingulate gyrus	6	34	16	4806	4.58
R Cerebellum	38	−80	−42	1141	3.91
L/R Occipital cortex	−28	−98	2	945	4.38
L Superior frontal gyrus	−16	34	52	789	3.90
R Temp/Occipital fusiform cortex	32	−52	−22	506	3.78
**LOW-WIN > HIGH-WIN**					
R Superior parietal lobule	38	−42	54	552	3.40

Z > 2.3, p < 0.05, cluster corrected. Temp, temporal; L, Left; R, Right.

We next examined BOLD signal change specifically due to receiving self-relevant gains in reputation by examining the outcome period in the description task, when participants discovered the words that they thought had been used to describe them. To conduct this analysis, we first sorted description trials into either High-positive or Low-positive groups based on the post-experiment word desirability ratings. This allowed us to make the social reward contrast in the description task analogous to the monetary reward contrast in the above card task. We next performed a self-relevant, social feedback interaction contrast (Self High-positive feedback > Other High-positive feedback) > (Self Low-positive feedback > Other Low-positive feedback). Similar to the monetary reward contrast, we found significant changes in BOLD signal in the ventral striatum (18, 10, −14; max *Z* = 4.32; *p* < 0.05, cluster corrected; Figure 3 and Table 2). For all brain regions that yielded significant activation clusters in the above contrasts, please see Tables 1, 2.

**Table 2:** **Significant activation clusters for self-relevant gains in reputation**.

**Region**	**MNI coordinates**			**Cluster size**	**Peak *z***
	***x***	***y***	***z***		
**(SELF HIGH-POSITIVE > OTHER HIGH-POSITIVE) >**					
**(SELF LOW-POSITIVE > OTHER LOW-POSITIVE)**					
L/R Precuneus	4	−46	50	11785	4.30
R Cerebellum	22	−38	−28	6553	3.93
L/R Ventral striatum	18	10	−14	1356	4.32
L Frontal pole	−26	40	42	1099	4.35
**(OTHER HIGH-POSITIVE > SELF HIGH-POSITIVE) >**					
**(OTHER LOW-POSITIVE > SELF LOW-POSITIVE)**					
None					

Z > 2.3, p < 0.05, cluster corrected.

### Region of interest fMRI: facebook intensity

We had an *a priori* hypothesis that the neural processing of positive social feedback regarding one's reputation in the nucleus accumbens positively correlates with the degree of Facebook use across individuals (see fMRI data analysis in Materials and Methods). To address this, we used each participant's Facebook intensity score (Figure 1) as a covariate in the group level fMRI analysis and performed the social feedback interaction contrast (Self High-positive > Other High-positive) > (Self Low-positive > Other Low-positive) within a mask of the bilateral nucleus accumbens. This analysis revealed a significant cluster of 22 voxels in the left nucleus accumbens (−8, 10, −10; max *Z* = 3.07; *p* < 0.05, cluster corrected for bilateral nucleus accumbens; Figure 3 Box).

We had also hypothesized that the nucleus accumbens response to monetary reward would not be related to Facebook use. To address this, we again used each participant's Facebook intensity score as a covariate and performed the monetary reward contrast (High-win > Low-win) within the nucleus accumbens mask. This analysis did not reveal a significant cluster.

To independently confirm and visualize these results, we performed correlation analyses using participants' BOLD signal change in response to both social and monetary reward within the nucleus accumbens and their Facebook intensity scores. First, we performed a conjunction analysis between the social feedback interaction contrast (Self High-positive > Other High-positive) > (Self Low-positive > Other Low-positive) and the monetary reward contrast (High-win > Low-win) within a mask of each side of the nucleus accumbens. To note, this conjunction analysis was performed independent of Facebook intensity score, therefore, we avoided a potential non-independence error. We then used the clusters from the conjunction analysis as functional region of interest masks (Figures 4A,B). From within these masks, we extracted parameter estimates of both the social feedback interaction contrast and the monetary reward contrast, and then performed correlation analyses with Facebook intensity scores. Parameter estimates from the social feedback interaction contrast within the left nucleus accumbens positively correlated with Facebook intensity scores (Pearson's *r* = 0.400, 95% confidence intervals = 0.147/0.615, *p* = 0.026; Figure 4C). This significant correlation was not observed in the right nucleus accumbens (Pearson's *r* = 0.107, 95% confidence intervals = −0.264/0.480, *p* = 0.565; Figure 4D). With regard to the parameter estimates from the monetary reward contrast, no correlation with Facebook intensity scores was observed in the left nucleus accumbens (Pearson's *r* = −0.250, 95% confidence intervals = −0.518/0.047, *p* = 0.174; Figure 4E) or the right nucleus accumbens (Pearson's *r* = −0.143, 95% confidence intervals = −0.492/0.224, *p* = 0.444; Figure 4F).

### Regression analysis with nucleus accumbens activity and personality measures

To confirm that individual differences in the left nucleus accumbens response are best explained by the degree of Facebook use rather than other personality traits, we performed a control step-wise regression analysis with Facebook intensity and the other self-reported personality measures (self-esteem, social desirability, narcissism, conformity and depression-related affect) to predict the parameter estimates from the social feedback interaction contrast within the left nucleus accumbens (these parameter estimates were used in Figure 4C). Our results demonstrate that Facebook use primarily explains our results [Adjusted *R*^2^ = 0.325, *F*_(2, 28)_ = 8.227, *p* = 0.002; Significant variables were: Facebook intensity, beta = 0.510; conformity, beta = −0.471] (see Materials and Methods).

## Discussion

Our primary research question investigated how individual differences in the neural processing of gains in reputation are related to differences in real-world reputation management, namely social media use. To this end, we employed Facebook use as a proxy of a real-world behavior aimed at reputation management. Our experimental results first confirmed previous research that both self-relevant gains in reputation and monetary reward are processed in the ventral striatum (Izuma et al., 2008). We then demonstrated that, relative to observing gains for others, the processing of gains in reputation in the left nucleus accumbens predicts the intensity of Facebook use across individuals. To note, nucleus accumbens activity revealed by the simple main effect contrast of Self High-positive social feedback > Self Low-positive social feedback, did not predict the intensity of Facebook use (see Contrasts to reveal gains in reputation in Materials and Methods). Furthermore, nucleus accumbens activity in response to monetary reward also did not predict Facebook use. Finally, our control regression analysis demonstrated that the activity in the left nucleus accumbens due to self-relevant gains in reputation is explained primarily by Facebook use.

Our individual differences result builds upon the research of others and extends the current knowledge of how nucleus accumbens function is related to human, reward-related behavior. To begin with, earlier animal studies have connected nucleus accumbens function to individual differences in reward seeking behaviors (Tõnissaar et al., 2006; Dalley et al., 2007). In addition, a relation between human brain structure and individual, reward-related personality traits has been established. For example, the stronger a person's white fiber tract connectivity between their ventral striatum and prefrontal cortex, the more likely they are to have a reward dependent personality (Cohen et al., 2009). Furthermore, it was recently demonstrated that individual differences in nucleus accumbens activity in response to pictures of food or sex predicts food consumption or sexual desire, respectively (Demos et al., 2012; Lawrence et al., 2012). In other words, the more sensitive a person's nucleus accumbens is to an image of food or sex, the more likely they are to attempt to obtain these things in the real world. Demos et al. state that their findings suggest a domain-general pattern (either food or sex) for reward-related brain activity whereby heightened activity in the nucleus accumbens may mediate appetitive behaviors. Our individual differences result corroborates this domain-general theory and extends it to a new domain by demonstrating that nucleus accumbens activity in response to self-relevant, social information predicts a real-world, self-relevant, social behavior. To note, an important feature of our study is that we provided participants with actual rewards in the scanner (the words they saw were taken as actual positive social feedback related to their character), as opposed to images of rewards (for example, pictures of food, which are not the same as eating the food in the scanner). Thus, our results indicate that the phenomenon of nucleus accumbens activity predicting behavior across participants is not only related to showing a picture of a reward, as in previous studies, but to the actual receipt of a reward.

To date, there has been very little neuroscientific research regarding social media use. One study examined brain structure as it relates to the number of Facebook friends (Kanai et al., 2012). Gray matter density in the temporal lobes and bilateral amygdala positively correlated with the number of Facebook friends. Amygdala volume has also been shown to positively correlate with real-world social network size in humans and animals (Bickart et al., 2010, 2012; Sallet et al., 2011). It was also shown that sharing information about the self, similar to posting a status update on Facebook or tweeting about yourself on Twitter, activates the nucleus accumbens (Tamir and Mitchell, 2012). Finally, another recent study demonstrated that Facebook use can evoke a psychophysiological state characterized by high positive valence and arousal (Mauri et al., 2011).

As mentioned above, we chose social media use, specifically Facebook use, because of its relation to reputation management. It should be noted however, that not all activity on Facebook is related to reputation. For example, people can read news from the site. We are not claiming that all activity on Facebook is reputation related and our research goal was not to dissect out the various aspects of Facebook use. As we described above in the introduction, much of the interaction on social media websites is in view of the user's friends or public. Thus, inherently, by using social media a person will experience an impact on their reputation. In addition, one of the most prevalent ways to socially interact on Facebook consists of users “liking” posted information. This approval is positive social feedback for the person who posted the information, and can be considered related to their reputation. In other words, if people are “liking” a Facebook user's posts, the Facebook user is viewed positively, and thus, has a good reputation. Furthermore, research has demonstrated that people use social media websites for impression management (Krämer and Winter, 2008; McAndrew and Jeong, 2012), and to maintain and increase their social capital (Ellison et al., 2007). Finally, books on reputation management now discuss the involvement of social media, especially Facebook, when managing one's reputation in today's society, suggesting ways in which reputation can be improved by using Facebook (Solove, 2008; Komisarjevsky, 2012). With all this in mind, we chose intensity of Facebook use, rather than another behavior, as our real-world reputation related behavior.

When designing our experiment we specifically included the “other” social feedback condition, and then analyzed our data accordingly with the social feedback interaction contrast. The other social feedback condition was included for two reasons. First, we wanted to isolate self-relevant gains in reputation. By performing the social feedback interaction contrast, we removed changes in BOLD signal associated with non-self-relevant gains in reputation (see Contrasts to reveal gains in reputation in Materials and Methods). Notably, a social feedback interaction contrast using self and other feedback conditions has previously been used to demonstrate social reward processing in the striatum (Izuma et al., 2008). Second, Facebook use has a social comparison component (McAndrew and Jeong, 2012). By including the other condition and performing the social feedback interaction contrast, we can observe changes in BOLD signal with respect to social comparison, between feedback for the self and feedback for others, across trials. Importantly, social comparison during the receipt of monetary reward has been shown to modulate ventral striatum activity (Fliessbach et al., 2007). With this in mind, it follows that we observed the relationship between nucleus accumbens activity and Facebook use specifically when examining the processing of gains in reputation for the self, relative to gains in reputation for others.

It should be noted that there are three possible limitations to our study. First, we have relied on a self-reported measure of Facebook use. A behavioral measure of Facebook use, which includes an actual assessment of Facebook activity, would improve upon our findings. Second, it could be that people who use Facebook more are more responsive to social feedback when received via a computer interface. To explain, although participants believe the positive social feedback is real (i.e., from other humans), participants received this feedback via a computer screen. Therefore, we cannot be sure that our individual differences finding concerning the processing of gains in reputation in the left nucleus accumbens will translate to direct human-to-human interactions. Third, because our primary research question concerned the relationship between gains in reputation and Facebook use, we did not include an “other” condition for the monetary reward card task. If we had included this condition and then subtracted out the change in BOLD signal due to observing another person receive monetary reward, it is possible that we would have revealed a relationship between self-relevant monetary reward and Facebook use.

Recent research has revealed some negative effects of social media. For example, Facebook interrupts productivity in schools and reduces grade point averages (Junco, 2011, 2012). Furthermore, reports of addiction to Facebook have started to surface (Kuss and Griffiths, 2011). Therefore, our findings relating individual social media use to the individual response of the brain's reward system may also be relevant for both educational and clinical research in the future. It's important to note, however, that our results do not determine if positive social feedback drives people to interact on social media, or if sustained use of social media changes the way positive social feedback is processed by the brain. Future longitudinal research may resolve this question of causality.

In conclusion, we found that the processing of self-relevant gains in reputation in the left nucleus accumbens predicts the intensity of Facebook use across individuals. This result was specific to positive social feedback for the self relative to observing positive social feedback for others. Furthermore, nucleus accumbens activity in response to monetary reward did not predict Facebook use. These findings extend our present knowledge of nucleus accumbens function as it relates to human behavior. Individual differences in the nucleus accumbens response to images of primary reward had earlier been shown to predict human behavior (Demos et al., 2012; Lawrence et al., 2012). We demonstrate that this previously established domain-general aspect of individual nucleus accumbens activity can be extended into the area of processing self-relevant social information, specifically in regard to reputation processing and real-world reputation management via social media.

### Conflict of interest statement

The authors declare that the research was conducted in the absence of any commercial or financial relationships that could be construed as a potential conflict of interest.
